# Evolutionary development of mother–child scaffolding for moral comprehension

**DOI:** 10.3389/fpsyg.2024.1397547

**Published:** 2024-10-16

**Authors:** Robert J. Beck

**Affiliations:** School of Education, University of California, Irvine, Irvine, CA, United States

**Keywords:** evolutionary development, ecological niche, narrative inquiry, scaffolding, harm/care, fairness/justice, moral comprehension, theory of mind

## Abstract

This evolutionary developmental study employed an experimental recursive narrative ecological niche, comparing scaffolded mother–child (5-year-old) pairs to unassisted controls as they independently viewed and discussed a realistic fictional family video depicting a father–daughter emotional conflict over the girl’s risky behavior, which violated harm/care and fairness/justice moral foundation norms. A microgenetic analysis was conducted on a selected variant pair that demonstrated high adaptive fitness in the niche by employing developmentally advanced cooperative scaffolding tools. The conversational ecosystem phase was characterized by repeated maternal theory-oriented “why” questions and coordinated child causal responses, forming a joint epistemic investigation that facilitated the child’s moral understanding of the characters’ responsibilities and motives. The pair used quasi-justice procedures to gather evidence, judge, and construct moral attributes for the characters. Their conversational mechanism was supported by mutual mindreading, mental time travel, and empathic communications, as they interacted simultaneously with each other and the story characters. A narrative ecological scaffolding theory emerged, establishing a standard for cooperative epistemic scaffolding between the mother and the child. Future training programs should utilize the Zone of Proximal Development method to instruct similar parent–child pairs.

## 1 Introduction

Evolutionary development engineering tools were employed to discover and develop linguistic mechanisms for the intergenerational scaffolding of moral comprehension norms (Tooby and Cosmides, 1992). The tools included an experimental narrative ecological system and the selection of a “smart variant” mother–child pair that demonstrated developmentally advanced fitness in adapting to the experimental conditions. The niche construction hypothesis proposes that hominins, animals, and plants alter ecological environments to make crucial features more salient and provide competitive advantages in obtaining resources (Sterelny, 2007, 2021). In this study, an experimental recursive narrative niche was predicted to support productive interactions between a movie, a contemporary narrative technology, and an ancestral oral tradition, mother–child conversation that made their shared situational schema more visible. Data from an experimental narrative ecology study was used to simulate an altered environment and track in real-time forms of naturalistic scaffolded joint attention instruction and computational processing: mother–child pairs (5 s) viewed a realistic fictional family video involving a child character’s breach of personal safety, then employed conversational scaffolding for supporting their children’s recall, who then individually retold the story (Beck and Clarke-Stewart, 1998; Clarke-Stewart and Beck, 1999). The naturalistic design enables an ontological study of moral “conflicts as they are recognized and dealt with as and when they arise” in a particular setting (Packer, 1989, p. 95). A smart variant pair was selected from the sample pool that responded with high adaptive fitness to the recursive ecological design in employing cooperative developmentally advanced scaffolding tools supporting the child’s moral comprehension (Mesoudi, 2008). The moral situations concerned evolutionary significant behaviors protecting children: cooperation, harm/care, and fairness/justice foundations (Curry et al., 2019; Graham et al., 2012; Haidt and Kesebir, 2010; Ditto et al., 2009; Fehr and Fischbacher, 2004). A narrative ecological scaffolding theory (NEST) guided a microgenetic study of the smart variant pair protocol and provided data explaining joint epistemic knowledge construction processes between the participants that generated their judgments of the movie characters.

In an approach commonly known as “evo-devo,” evolutionists, historians of science, paleo-anthropologists, primatologists, philosophers, and cultural developmental psychologists theorized an evolutionary approach inquiring into the intergenerational transmission and development of culture (Levins and Lowentin, 1985; Wimsatt and Griesemer, 2007; Odling-Smee and Laland, 2011; Godfrey-Smith, 2001; Tooby and Cosmides, 1992; Sterelny, 2007; Corballis, 2011; Skyrms, 2003; Shweder and LeVine, 1984; Tomasello, 1999; Ramstead et al., 2016; Stotz, 2014; Caporael et al., 2014). Rather than asking how human behavior evolved under natural selection, this approach selects features and population resources critically important to evolutionary dynamics (Godfrey-Smith, 2001). In human evolution, the usual relationship between organism and environment has virtually reversed in adaptation. Cultural invention has replaced genetic change as an effective source of variation. Consciousness allows people to analyze and make deliberate alterations; thus, adaptation of the environment to the organism has become the dominant mode (Levins and Lowentin, 1985, p. 69). The modification of the environment variably affects the fitness of the constructing mother and child agents in the current context. Such agents change as a function of the fitness of their constructed artifacts (Wimsatt and Griesemer, 2007, p. 247).

Inheritance is hence defined as the parental transfer to the next generation of all the developmental resources, including but not limited to DNA, that permit the reconstruction and modification of the developmental system. This developmental system is the whole organism-developmental niche complex. This reconstruction and modification encompass developmentally entrenched effects and sources for the expression of novel phenotypic variation (Stotz, 2014, p. 5).

Niche construction theory reunites evolutionary theory with ecological theory, in which language is considered an integrated biocultural system comprising the *semiosphere* that interacts with the *technosphere* material culture (Sinha, 2015). The present narrative ecological model proposes that evolutionary development involves the semiosphere, in which parent–child conversation interacts productively with the technosphere, e.g., family movies, to model an innovative scaffolding approach for children’s narrative and moral development (van de Pol et al., 2010; Shvarts and Bakker, 2019).

An engineering approach to evolutionary analysis advises the selection of a pattern of behavior that comprises an innovation in an ancestral environment and combines it with a successful contemporary adaptive target and performance evaluation of that behavior (Tooby and Cosmides, 1992; Ackerman et al., 2012). In this study, the innovative ancestral environment entailed moral behaviors including cooperation, harm/care, and fairness/justice moral foundations theorized as emerging in *Homo sapiens*, ca 200-50kya (Tomasello, 1999). A successful contemporary investigation was selected as an adaptive target in which a narrative ecological niche formed a demanding experimental task (Beck and Clarke-Stewart, 1998). A sample of 63-month-old children, SD.2 (*n* = 31), watched a 5′ videotaped segment from the movie Prancer (1989) that involved realistic fictional father-daughter (7–8 years) moral conflicts over the girl putting herself in danger and the father intending to put an injured deer out of its misery. During the ensuing conversation these events aroused a cluster of moral behaviors; each participating child then retold the movie. A comparison group (*n* = 14) watched the video with their mothers but did not discuss it. The children in the mother-scaffolded discussion group had both better recall of objective actions (*t* = 3.41, *p* < 0.001) and comprehension of characters’ internal states in the story (*t* = 4.38, *p* < 0.001). Other findings relevant to the present study showed that the number of questions (*p* < 0.01), extended exchanges (*p* < 0.01), correction of mistakes (*p* < 0.01), and use of character emotion words (*p* < 0.05) in the conversation significantly correlated with the recalled comprehension of the characters’ internal states in the retold stories (Beck and Clarke-Stewart, 1998).

## 2 Materials and methods

### 2.1 Study participants: selection of a mother–child *smart variant* pair

Although evolutionary scientists emphasize universal patterns characterizing all members of a species, an evo-devo approach is also concerned with how individuals adapt their behavior to their particular life circumstances (Grotuss et al., 2007, p. 450). An experimental niche complex could promote evolutionary change by discovering particular mother–child agents who exhibit advanced fitness in response to altered environments and analyzing their inventive communication methods. The variants that occur in genetic evolution are random. However, those in ontogenetic processes are not. Some organisms may display evolved aptitudes and capacities for learning.

Experienced others, such as parents, are a reservoir of *smart* var*iants*, allowing naive individuals to shortcut the many iterations of ontogenetic selection necessary to learn for themselves behavioral patterns appropriate to their environment and thus leapfrog to the functional and already-tested solutions established by others (Mesoudi, 2008, my emphasis).

Learned knowledge cannot be genetically inherited, but evolved agents “can guide niche construction, the consequences of which can be inherited through ecological inheritance”.

### 2.2 Examining a sub-sample of successful mother–child pairs

From an evolutionary perspective, “evolved features” need to be demonstrated in at least one subpopulation in an ecosystem (Uller and Helanterä, 2019). To better understand the variations in mother–child joint attention communications and to discover/select a smart variant pair, we initially analyzed a sub-sample of 13 pairs in the experimental study, 42% of the experimental group (*n* = 31), that had *extended exchanges* of 5–45 turns on critical topics of the story. A quantitative analysis of emotion words in the sub-sample was conducted to determine the frequency and variability of anger, fear, and sadness emotion mentions, signifying that the participants referred to characters’ mental states. The results were that these 13 discussions contained a total of 134 sentences that contained emotion words. Notably, the unassisted group had *zero* words for emotions or subjective states in their retellings. Within the experimental extended dialogues, for anger, there was a total of 33 sentences contained within 10 pair exchanges; for fear, there were 10 sentences contained within four pair exchanges; and for sadness, there were a total of 91 sentences in the 13 pair exchanges.

### 2.3 Selection of the smart variant mother–child pair

The investigator then reviewed seven sets of pair-extended exchanges that addressed the *moral issues* in detail: the girl putting herself at risk and the father intending to shoot the hurt deer (Beck and Bear, 2002). *Only the selected smart variant pair* (SV) addressed a pattern of behavior of the highest evolutionary significance: the safety and protection of offspring (Burkart et al., 2018; Griffiths and Gray, 1994). Only the SV pair approached the movie as an opportunity to teach and/or reconfirm the principal moral norms of the movie story by attributing harm and care to the characters’ behaviors and inducing the child to enact procedural justice roles in judging them (Graham et al., 2012). All the other extended discussions focused solely on the moral issue concerning the deer, the question of putting a hurt animal out of its misery, and the parental solution, which, in the movie, was to shoot the deer. No child in this sub-group could comprehend this rule or accept this solution. The numerous empathic expressions of fear and sadness in those exchanges concerned the children’s feelings for the animal and the girl character’s frustration in helping it. Only the SV mother employed multiple why questions calling for her child’s explanations of the characters’ behaviors, questions that also enabled the boy to share her mind. The mother positioned her son to identify with the characters and view the story from their perspectives. On this basis, we selected the SV pair for microgenetic analysis. In using this study to provide empirical support for understanding the complexities of transmission of cultural inheritance, the investigator, as an experimenter-evolutionary agent, collaborates with an exceptional mother–child pair who adapted naturalistically and with high fitness to the demanding, problematic, and enriched conditions of the experiment.

### 2.4 Narrative ecological scaffolding theory

To guide a microgenetic analysis of the SV pair’s communications in the experimental ecology, a narrative ecological scaffolding theory (NEST) was developed from research in child development, neuroscience, and evolution. Scaffolding was aptly described as “a theoretical model of the teacher in informal education” (Greenfield, 1984, p. 118). At the time of the experiment, the mother in the SV pair had been the continuous informal teacher of her child for 5 years. As children are restricted in their mobility and participation in the adult world, stories are their principal source of access to the situations in the possible worlds they will encounter in adult life. In the “Narrative Practice Hypothesis,” Hutto (2007, p. 53) claimed that ordinary folk storytelling practices provide the crucial training set needed for children to understand *in situ* the reasons for familiar partners’ thoughts, feelings and actions in appropriate contexts and backgrounds and consider current circumstances and history. “Most children are not only repeatedly exposed to such stories, but normally this occurs in a very rich setting, with engaged participants on both sides,” and children are involved during such dialogues to be prompted at crucial points to offer their own explanations; “they are invited to apply, demonstrate and extend their prior understanding” (Hutto, 2007, p. 53). Moreover, narratives are useful in simulating the goals and obstacles of everyday survival as they also emerge in ancestral environments (Sugiyama, 2001).

Although he never used the metaphor, most theories of scaffolding are beholden to zone of proximal development (ZPD) of Vygotsky (1978), arguing that children learn to solve a problem, carry out a task, or achieve a goal that would be beyond their unassisted efforts through a temporary support system of interactive and contingent social exchanges, such as the findings in the authors’ study—extended exchanges, multiple questions, correction of mistakes, and use of emotion and other mental state words. Among other frequently employed maternal scaffolding strategies that have been theorized, it is worth noting that the use of modeling, direction maintenance, hinting, and reduction of degrees of freedom, i.e., simplifying to reduce cognitive load and transfer of responsibility or fading, affords children’s internalized understandings (Wood et al., 1976; van de Pol et al., 2010).

Scaffolding has been the focus of extensive research, generating over 1,000 studies in the last decade alone (Shvarts and Bakker, 2019). It is often explored through maternal interventions in conversational settings, particularly in relation to young children’s narrative competency in creating or retelling stories. By representing norm-breaking events, narratives necessitate reflection and analysis, requiring us to interpret and make meaning of experience (Nelson, 2003). The internalization of narrative forms enables individuals to understand their interface, or mental model, of internal and external linguistically represented worlds in which motivated human agents act in specific physical environments to achieve goals, thus coming to understand the differences between one’s own mind and others’ minds (Herman, 2007; Hutto, 2007 in Nelson and Fivush, 2020, p. 80). As such, a narrative is a powerful tool for socialization (Miller et al., 1997), an effective way to transmit cultural knowledge, values, and beliefs (Campbell, 1988; McKeough et al., 2008, p. 150), and in the perspective of this experiment, narrative competency is a central mental and linguistic joint attention scaffolding tool for mothers and children alike.

In the present ecological design, the movie narrative comprises a cultural material object providing an external resource.

Structurally, a scaffold is a supporting framework … that supports… materials, tools, agents, and processes as when builders use scaffolding to support workers and their materials while erecting or repairing a building…Some of the developmental machinery is internal, cognitive, affective, conative, and normative…but large chunks of it [culture] are at least partially external (Wimsatt and Griesemer, 2007, p. 229, 260).

In experimental ecology, the external information consists of “patterns of variation in cultural items [that] have a direct path of influence” (Richerson and Boyd, 2004), such as the characters’ behaviors in the movie moral conflict scenarios and the technology of close-ups, edits, music and other sound effects used to heighten the viewers’ and characters’ emotions (Rooney and Bálint, 2018). Participants’ mental models of narratives also function as internal scaffolds for guiding learning.

### 2.5 Text comprehension: narrative situational models

A narrative situation model is a mental model or representational schema constructed by agents, whether as actors, readers, or viewers, to comprehend situations in the real world or texts.

When humans perceive the world, vision yields a mental model of what things are where in the scene in front of them (Marr, 1982). Likewise, when they understand a description of the world, they can construct a similar, albeit less rich, representation—a mental model of the world based on the meaning of the description and their knowledge (Johnson-Laird, 1983) in Johnson-Laird (2010).

Models consist of standard properties of stories investigated in text comprehension research: protagonists’ attributes, goal directions, and affects/motivations as they occur causally in space/setting and time (Graesser et al., 1997; Graesser et al., 2002). Narrative competency is paramount in enabling mother–child communications about social events and a mutual understanding of how the world works. Schemas refer to memory structures that store pre-constructed world knowledge of phenomena that represent probable situations in order to recognize and classify the developing patterns an agent faces in the various worlds in making adaptive goal-directed decisions (Derry, 1996). While it is unlikely that the SV mother’s and child’s situation models were equally developed, the analysis will show that the pair’s models were highly coordinated: “If two or more people are required to communicate about a situation, they must each construct a similar mental model of it” (Derry, 1996, p. 168). We will argue that an important outcome of the SV mother–child information processing was the development of the child’s mental model of the movie, particularly its moral issues and the pair’s joint mental model.

Using these mental models, viewers and discussants generate knowledge-based *inferences* during reading or viewing stories, such as

goals and plans that motivate characters’ actions, character traits, characters’ knowledge and beliefs, character emotions, causes of events, the consequences of events and actions, properties of objects, spatial contexts, spatial relationships among entities, the global theme or point [moral] of the text, the referents of nouns and pronouns, the attitudes of the writer, and the appropriate emotional reaction of the reader (in Graesser et al., 1997, p. 181; see also Trabasso et al., 1992; Labov and Waletsky, 1967).

Knowledge-based inferences are made to fulfill reader/viewer goals to construct meaningful organization of the material, e.g., integrating local chunks of information into higher order concepts, e.g., extract a moral, and/or to explain why, i.e., to create psychological and causal theories (Graesser et al., 1994, p. 371). As applied to moral comprehension, therefore, narrative competency consists of a set of abilities in which one can represent an agent’s progress toward a goal, provide a sequential order of events, note violations of the canonical, indicate cultural norms, and include the narrator’s perspective as well as viewing the story from the perspective of the protagonists (Bruner, 1990, p. 77; Mar, 2018b). NEST will test the constructivist theory hypothesis that the most important inferences are those that “explain *why* events, actions, and states occur” (Graesser et al., 1994, p. 183).

The overlap between text comprehension and neuroscience situational mental model research frameworks is considerable and increasingly, comprises both external material elements and internal states. Neuroscience studies have confirmed that specific brain regions are activated during comprehension tasks that attend to the situation models discovered by text comprehension researchers.

[T]ypically, situations occur in a *physical setting*, with *agents* present (at least oneself and often others), performing *actions on objects* (including people) to produce desired outcomes. Not only does the physical world contribute elements to a situation, but so does the rich milieu of internal experience, including self-relevance (e.g., *goals, values, norms, identities*), which initiates *emotion* and *motivation*, often accompanied by various forms of *mentalizing* (e.g., evaluation, prediction) (Barsalou et al., 2018, p. 2, my emphasis).

Neuroscience research also confirms that situational information is constructed from classes of inferences concerning the goals and plans that motivate characters’ knowledge and beliefs, traits, emotions, the causes of events, properties of objects, spatial relationships among entities, and expectations about future episodes in the plot. Some of these inferences are generated online, e.g., during viewing a movie, while others are constructed offline during retrieval, e.g., during a conversation evaluating the moral issues in the movie.

### 2.6 Conversation: the epistemic triangle

The mother–child conversation is assumed to be the scaffolding apex of the social epistemic developmental process in the current ecological niche construction. The locus of integration of subject-object and social interaction “consisting of an active subject, the object of knowledge, and a (real or implicit) interlocutor, together with their mutual relations…was termed the epistemic triangle” [Chapman, 1991, p. 211; Carpendale and Lewis, 2004, p. 84–85; see also Vygotsky’s (1978) related concept of the “mediational triangle” that leads to internalized relations with objects]. In the current study, the active subject is the Kindergartner, the movie is the object of knowledge, and the real interlocutor is, of course, the mother, who occupies the apex of the triangle in referring simultaneously to her child and the characters and events of the movie. The epistemic triangle is a model based on research in child development theorizing the cultural origins of human cognition as originating in mother–child joint intentional-causal communicational representations of cultural objects and events (Tomasello, 1999). From the first days of childhood, the epistemic triangle processes are constructed through mother–child references to objects in the world. In the present usage, the epistemic triangle is conceived as a scaffolding mechanism whose objective is the joint mother–child construction of children’s moral comprehension through interactive conversational strategies directed toward explanations of events in the movie, a cultural object they have viewed, but naturally, in which they have had no direct involvement.

### 2.7 Moral development and comprehension

Responding to the security issues in the movie, our theoretical model of moral comprehension development is informed principally by harm/care, cooperation, and fairness/justice moral foundation theories (Graham et al., 2012; Kohlberg et al., 1983), but we will show that these moral foundations also integrate process-relational (Carpendale et al., 2021) and domain-specific (Turiel and Banas, 2020) theories. It is likely that judgments made using moral and social domain principles coexist in early childhood (Laurenco, 2014, p. 2). In process-relational approaches, children’s moral sense is grounded in activities conducted in mutual relational social contexts, initially in mother–child and other parental and caregiver dyadic relationships and later in interactions with peers. Children’s protecting/caring behaviors emerge in early development and continue to develop in preschool (Geraci et al., 2023). Children form moral judgments about right and wrong at an early age, which include understanding the need to avoid harm and benefit people (Smetana et al., 2018; Smetana et al., 2014). Caring for others, which children value as ends in themselves, is foundational in structuring the linguistic and emotional interactions during which constituted rules emerge. The participating child’s moral judgments were structured in this study by referencing interactions of the characters in the movie in which the young girl putting herself in harm’s way caused pain and negative judgment by her father. Moral rules were instructed to understand the dynamics of the mutual relational context of the father-daughter pair.

Domain theories distinguish between moral, conventional, and personal domains, holding that rules concerning conventional activities (do not eat with your fingers) and those concerning morality (do not harm others) depend on reasoning based on freedom of choice and independence, particularly in individualistic rather than collectivist cultures. Domain-specific rules are up to the individual as long as they do not harm others (Turiel and Banas, 2020, p. 28) and have been characterized as involving rational decisions. While the particular socioemotional domain is important in affording and constraining relevant conventional issues, recent domain theory concerning differences between moral and social domains concludes that rules may be mediated by using multiple standards in coordinating moral judgments (Turiel and Banas, 2020).

The group of foundational moral systems to be analyzed in this study are innate, highly entrenched intuitive modules, including cooperation, harm/care, and fairness/justice mental modules. These are directed by intuitive, affective behaviors and shaped by long-term evolutionary and cultural processes (Graham et al., 2012). The systems have been theorized to make cooperative social life possible (Haidt and Kesebir, 2010, cited in Curry et al., 2019, p. 106) and have been validated through questionnaires, now in a second generation, MFQ-2, using structural and factorial modeling (Zakharin and Bates, 2023) as well as external validity measures (Atari et al., 2023).

*Cooperation* has been identified in evolutionary theory and moral development as a primary foundation involved in coordinating social behavior. Cooperation is operated in the SV protocol through the mother–child pair’s conversational mechanisms that serve to productively scaffold harm/care and fairness/justice moral systems to support children’s moral comprehension. Reciprocal responses during dyadic linguistic turn-taking interactions are a source of the expected epistemic benefits for children (Fernyhough, 2008). Fairness, i.e., equality of distribution accorded to self and others, emerges in the first 2 years of child development (Geraci et al., 2023). Although there are variations dependent on research methods and participants, e.g., human vs. animal and developmental age, cooperation may be defined as reciprocal joint behavior involving helping and sharing directed toward goals providing mutual benefits (Curry et al., 2019; Batson et al., 1995; Komorita and Parks, 1994; Warneken and Tomasello, 2006; Smith and Warneken, 2016). A foundational component of human declarative communication is the development of children’s enjoyment in participating in interaction as a goal (Carpendale et al., 2021, p. 6).

The *harm/care* foundation evolved to protect and care for children and group members. As early as the first 2 years of life, researchers have shown that children demonstrate abilities to evaluate and foster prosocial behaviors such as distributive, affiliative, sharing, and helpful behaviors and a tendency to punish antisocial agents and expect bystanders to do so (Geraci et al., 2023). “Researchers from diverse perspectives concluded that, by the preschool years, nearly all children share the core moral belief that intentionally harming innocent others is categorically wrong” (Arsenio and Lemerise, 2004; Gasser et al., 2012; Nunner-Winkler, 2013 in Jambon et al., 1344). Emotions play a key role in this moral foundation, for social norm-breaking is signaled by visual and auditory expressions of suffering or distress and often involves anger at perpetrators of harm.

[T]he sudden appearance in consciousness, or at the fringe of consciousness, of an evaluative feeling (like–dislike, good–bad) about the character or actions of a person, without any conscious awareness of having gone through steps of search, weighing evidence, or inferring a conclusion (Haidt and Bjorklund, 2008, p.188, modified from Haidt, 2001; cited in Graham et al., 2012, p.11).

Specific caregiver emotional reaction to a moral transgression involving harm plays a key role in socializing harm/care norms (Carpendale et al., 2021). Parents frequently express pain, anger, and seriousness in their reactions to children’s moral transgressions (Dahl et al., 2013; Dahl and Campos, 2013, cited in Essler and Paulus, 2022, p. 17). More generally, social interactions and responses to transgressions facilitate the use of moral reasoning in the construction of moral understanding that builds on predispositions that are evident in infancy (Geraci et al., 2023, p. 01; see also Yoo and Smetana, 2022). Essler and Paulus (2022, p. 7) supported these assumptions that parents, by reasoning with their children about the needs, emotions, norms, and consequences involved in morally relevant situations, support children to process and elaborate on moral transgression and its effect on others and consider alternative behaviors.

It is assumed that the *fairness/justice* moral module evolved culturally to develop characteristics resembling procedural justice institutions such as law enforcement and the court system that involve motivated reasoning and consideration of relevant rules.

Reasoning is more like arguing than like rational, dispassionate deliberation (Mercier and Sperber, 2011), and people think and act more like intuitive lawyers than intuitive scientists (Baumeister and Newman, 1994; Haidt, 2007, 2012 in Ditto et al., 2009, p.11).

Unlike scientific reasoning, a lawyer or detective argues to prove a pre-existing conclusion that a perpetrator, for example, has violated a law and brings evidence to support that theory. We hypothesize that cooperative turn-taking and justice procedures provide cultural and linguistic methods and procedures for the smart variant of children’s harm/care moral development.

### 2.8 Method: a microgenetic study

A salient criticism of research in narrative scaffolding is that “surprisingly little attention has been devoted to specifying the meaning, function, and mechanisms of the construct” (Svane et al., 2021, p. 4). The assertion is that not enough research has been directed to the joint attentional reciprocal processes of mother–child discourse. The epistemic triangle was designated as the scaffolding structure in which mothers and children construct knowledge of events; however, the joint mechanisms and processes through which theorized comprehension processes are shared, and combined must be tested in the ecosystems in which they occur. Fundamental to conversation analysis is painstaking turn-by-turn interpretation of participants’ reciprocal talk, presuming the need for qualitative and hermeneutic re-readings of transcripts (Schegloff, 2006). The search should be for the internal mental and external functioning structures that lead not to conformity with the demands of the normative cultural context but to the emergence of “novel mechanisms in ways *coordinated with* context demands” (Valsiner, 1996, p. 47, author’s emphasis).

In contrast, the quantitative coding of transcripts, unsurprisingly the common method in scaffolding studies, is typically used to measure the frequency of parental open-ended questions and other indicators statistically related to the frequency of children’s memory information. However, such quantitative analysis masks “the *mechanisms* of how the specific parental utterance types *directly* affect children’s memory information within the conversation, and how children’s utterances are directly responded to by the parents, remain unexplored” (Svane et al., 2021, p. 8). For example, parental repetitions and children’s memory information may downplay the value of repetitions due to the traditional analysis method where all parental repetitive utterances are collapsed into a composite score. These methodological criticisms lead us to favor a microgenetic study involving the intensive analysis of the SV mother–child pair whose participants employed the scaffolding techniques reported in our original research, e.g., questioning, extended exchanges, etc., as well as utilizing other scaffolding mechanisms, e.g., the external movie affordances and internal situational models that have been delineated.

### 2.9 Ecological niche working hypothesis

The experimental ecological niche tests that the hypothesis of increased cognitive complexity leading to evolved scaffolding mechanisms depends on productive, interactive feedback loops between recursive ecosystems. In this case, historically recent technological innovations engineered by humans, realistic fictional family videos, *ca* 50 years interact with the pre-historic oral mechanisms of social intelligence, parent/caregiver-child conversational language. The tri-level recursive structure consists of different forms of the same narrative, *Prancer,* that are sequentially, topologically, and reciprocally connected by the demands of the experimental memory and comprehension task. The reciprocal structures conform to Bronfenbrenner’s (1977) model of experimental ecology. The cultural exosystem movie story furnished a resource used by the SV mother and child co-viewers, whose perceptual sensory-motor and emotional experiences and memories of the movie were subsequently utilized in the family mesosystem conversation and, in turn, provided mother–child constructed knowledge for use when individual children retold the cultural story. The present experimental narrative ecological niche, therefore, is a retrospective, recursive co-construction in which the participants sequentially share and integrate their internal memory, affects, and moral comprehension of a material perceptual experience, the movie, through external conversational mechanisms that introduce semantic lexicon transformations of the participants’ episodic memories (Corballis, 2011). The niche is topologically structured in that the phenomena at one level are contained, albeit cognitively reorganized, at other levels. The experimental approach aims to *disturb an existing balance between the levels*, and the expected main effects are *interactions, such as* var*iations in family conversations*. In a recursive ecology, the conversation is expected to abstract and simplify the movie story while introducing complexity. The principal conversation mechanism involved in such reorganization is turn-taking, which emphasizes causal explanations.

Memory for information is enhanced when the reader/viewer constructs causal explanations of why events in the situation model occur and why the writer expresses information (Chi et al., 1994; Graesser et al., 1994; Pressley et al., 1988; Trabasso and Magliano, 1996; Zwaan and Brown, 1996). Readers actively seek these explanations during reading (Graesser et al., 1994) (Graesser et al., 1997, p. 175).

## 3 Narrative ecological scaffolding: a family movie

*In the opening scene, Jessica, an 8–9-year-old girl, is seen following an animal’s tracks and hears shots as she walks through snowy fields and forests. Cut to Jessica’s father coming across his daughter unexpectedly while driving his truck on a forest road to go shopping. He angrily criticizes her for being in the forest alone, a repeated misbehavior: “A hunter could shoot you,” he shouts. She explains that she was looking for Prancer, the eponymous Santa reindeer. They then have a tearful confrontation when her father tells her he is thinking about sending her to live with her Aunt Sarah because he is unable to give her the things she needs now that her mother is no longer there. In close-up, Jessica yells to her father to stop, and the truck screeches to a halt as Prancer suddenly appears on the road in front of them, his leg bleeding. The father goes to get his gun to put the animal out of its misery. Jessica tries to stop him. “No, Daddy, no!” They turn around, and the animal mysteriously disappears (Prancer, 1989).*

### 3.1 Interpretation of the movie

While this movie is family entertainment and includes a magical element, a famous era critic described it aptly.

The best thing about “Prancer” is that it does not insult anyone’s intelligence. Smaller kids will identify with Jessica’s fierce resolve to get Prancer back into action, and older viewers will appreciate that *the movie takes place in an approximation of the real world* (Ebert, 1989, my emphasis).

Typical of realistic fictional movies, *Prancer* “assumes an isomorphism between representations of narrative and real-world events. For example, narrative events are causally linked within a narrative time and space, in much the way that we understand real-world events” (Magliano et al., 2007, p. 379). This movie provided material scaffolds that afforded participants issues to remember and possibilities for subsequent discussion to help their children morally comprehend and retell the story. The agents’ variability in performance is a function of their abilities and preferences in using the *affordances* and *constraints* of the ecological niche resources at the different levels (Ramstead et al., 2016, p. 5–6).

### 3.2 The moral situation and characters: breach of the harm/care and fairness/justice norms

In this context, an important source of sociocultural knowledge-building discourse potentially takes place when caregivers, acting as “moral guardians,” deal with “situations of accountability,” that is, norm-breaking events involving any departure “from what we consider ordinary expectable or approvable behavior” (Much and Shweder, 1978, p. 21; Shweder and Much, 1991; Labov and Waletsky, 1967). The implication is that the communication of sociocultural information is best understood and adapted for instruction by investigating naturalistic norm-breaking situations in which parents use *accountability* methods in emotionally conflicted situations.

The daughter’s search for the reindeer in a forest during hunting season creates a dangerous norm-breaking moral situation in which harm/care and fairness/justice values apply. As children become ambulatory, a vital context for supporting survival arises in prudential security situations in which children may place themselves in harm’s way by dint of their exploratory drives and lack of knowledge of dangers in the world (Smetana, 2017). When such breaches of norms come to the attention of parents and caregivers, holding children accountable frequently ensues. Nevertheless, there is no moral disciplinary instruction, *per se,* followed in the movie. As a resource, the movie allows mother–child pairs to use the events to teach/confirm the relevant sociomoral principles and assess their children’s knowledge thereof in the conversation.

### 3.3 Identification of movie characters and transportation into the narrative: film technology

The movie intentionally directed young viewers’ attention by placing both a child and a deer in danger and thus aroused their caring, empathic emotions toward those characters. “A major affordance offered by conventional movies is empathy with characters” (Tan, 2018, p. 4). The relationship between the viewer and a film story has been conceptualized as supporting identification with the characters and transportation into the narrative. Identification with characters is closely associated with empathy and entails perceiving, understanding, and emotionally responding to characters’ feelings (Zillmann, 1991). Bordwell (1985) argued for the importance of agent-based schema in making sense of movies by focusing on characters’ cultural roles, in the present case, parent care-giving responsibilities, children’s testing of norms, and the instruction of personal moral characteristics. Transportation evokes associations with travel into the film’s story-world and is associated with an amplification of the emotional quality of the experience (Green and Brock, 2002). While there is a relative lack of research using film stories for developing social skills, an alternative source of information rests on the findings that shared narrative readings of picture books between mothers and children have enabled pre-literate children to simulate social worlds (Mar, 2018a,b; Kucirkova, 2019; Nikolajeva, 2014b; Oatley, 1999; Mar and Oatley, 2008; Melzi et al., 2011) and enter a “community of minds” (Nelson, 2003). Like all film viewers, the experimental study participants, including the SV pair, were subject to a film-induced emotion that made them aware of being in the middle of the story world as a witness to events befalling characters. In most movies, and *Prancer* specifically, the characters communicate emotions directly without the narrator’s assistance. The technology is powerful in manipulating emotions, particularly the viewer’s sense of danger is critical to the enhancement of harm-care situations through sound, lighting, music, and timely edited close-up shots of the protagonists (Rooney and Bálint, 2018).

## 4 Narrative ecological scaffolding: mother (M) and kindergartner (K)

T1. M: And why was the daddy angry?
T2. K: Cause she was wandering all around?
T3. M: Okay, and was he angry in a bad way or a good way?
T4. K: A bad way.
T5. M: Why was it bad?
T6. K: Cause he was yelling at her.
T7. M: Do you know why he was angry? … Why was he angry at her for wandering around?
T8. K: Cause she wasn’t supposed to.
T9. M: Yeah. Why? What could happen?
T10. K: She could have got shot.
T11. M: Okay, he was angry because he was…?
T12. K: Because she could have got shot.
T13. M: Uh huh, how was he feeling? Cause he was…
T14. K: …Mad.
T15. M: At the girl?
T16. K: At the girl?
T17. M: No.
T18. K: The reindeer!
T19. M: No, no, no. He was angry because he was afraid, right? What was he afraid of?
T20. K: Afraid she was gonna die.
T21. M: Afraid she was gonna die? Yeah. Well, yeah, he was afraid she might get hurt wandering around in that snow, right?
T22. K. Wait, right?
T23. M. Right.
T24. M. And what was she afraid about?
T25. K. That she was gonna, that he was gonna kill the deer. That, that she was yelling. That he was yelling at her.

### 4.1 Interpretation of the SV protocol

#### 4.1.1 Modified I/RE turn-taking code

The SV conversational interaction was characterized by standard reciprocal turn-taking in which the pair used linguistic action chains and sequences (Levinson, 2006, p. 44). This turn-taking mechanism was most likely based on patterns of institutional academic discourse acquired by the participating mother in her formal schooling. From K-12 to university postgraduate education, questioning is a ubiquitous technique practiced in relatively formal sequences compared with ordinary dialogue. According to Wells (1999) and numerous others, teachers’ narrative inquiry (I) or questioning moves, are followed by students’ responses (R), and this, in turn, is usually followed by teachers’ evaluations (E), although there is evidence that the sequence is more commonly IRF, *feedback* or *follow-up* of all kinds in the third turn (Sinclair and Coulthard, 1975; Lemke, 1990; Mercer, 2002). What is innovative in the present usage of the IRE turn-taking is that K was given responsibility for both the response, R, and evaluation, E, i.e., he participated in an I/RE code. Thus, M’s power in assuming she has the authoritative answer as a teacher and parent is here transferred in part to K, whose repeated evaluations support the view that moral comprehension scaffolding involves the induction of the child’s epistemic judgments, which, as we will see, are applied to the story characters. However, the record shows that in one exchange (T19), M could still contradict her son’s evaluation.

Initial interpretation of the conversational record begins with methods drawn from the disciplinary fields of conversation analysis/discourse processes/narrative inquiry that prioritize close reading of the cooperative turn-taking structure to determine grammatic/semantic generated meanings, including repeated analyses of each speaking turn, adjacency pairs, sequencing, and inferencing/comprehension processes (Schegloff, 2006; Sacks et al., 1974; Graesser, 2002).

#### 4.1.2 An entrenched conversational mechanism for cooperative moral socialization: why/because of grammatical turn-taking

At the forefront of the SV mother–child cooperative joint participation was the productive use of M’s recurring *why* questions concerning F’s angry emotion and K’s responses, providing evidence of J’s causally linked transgressive actions. M asked seven *why*, or close variations of explanation-inducing questions to inquire into K’s moral reasoning for the father’s anger: “Why was daddy angry?” (twice), “Why was it bad anger?” “why, what could happen?” “angry because he was?” “how was he feeling, cause?” and “what was he afraid of?” Her questions are answered by K with reference to the security-violating *actions* of J with causal responses—“cause she was wandering around,” “cause he [F] was yelling,” and “cause she [J] wasn’t supposed to.” And importantly, in M’s question about J’s behavior, “What could happen? K states, “She could have got shot” (twice), and goes further, “She was gonna die.”

Mother and child appear to have employed entrenched, reciprocal joint attention “why-because” grammatical linguistic patterns of interactive communication to induce the boy’s comprehension of the movie (Caporael, 1997). It is assumed that “after comprehending a text, one might reasonably be expected to answer questions about it, recall or summarize it, verify statements about it, paraphrase it, and so on (Kintsch, 1988, p.163). M uses these why-because forms both to assess K’s moral comprehension and to assure that mother and son agree, as well as to expose disagreement on the facts and reasoning of the case against J. During most of the 25 turns, as M revealed *her mind* through questions that directed attention to *her memories* and *evaluations* of J’s behavior, K answered and completed her communications by revealing *his memories* and *evaluations* of the movie characters. Her use of repeated why questions induced K to repeatedly deduce, that is, conceptualize his understanding of the father-daughter relationship in the dangerous situation, and the same questions found both parties building and elaborating subjective and objective characterizations of these characters while simultaneously finding the girl guilty of misconduct.

Two implications are emerging from these discourse processes: first, these coordinated turns revealed an entrenched, well-practiced, synchronized mother–child practice in which there was “an ability to simulate the other’s point of view but also to imagine what he or she thinks your point of view is,” indicating that the child has an adequately developed folk theory of mind (ToM) (Levinson, 2006, p. 49). ToM is an extensively researched capability to mindread others’ mental states in order to explain and predict their intentions and likely actions (Perner, 2000; Astington and Pelletier, 1996; Premack and Woodruff, 1978); second, the use of why questions and coordinated responses constituted explanatory discussion involving moral comprehension.

According to the assumption of explanation, why questions are fundamental questions that drive comprehension. The strong version of this assumption is why questions drive the comprehension of all text genres, not just narratives. Stated differently, explanation-based reasoning is an invariant feature of all comprehension, whether the input is narrative, expository, film, or physical and social activities in everyday life (Graesser, 2002, p. 23).

M’s questions about F’s anger directed K through a chain of inductive inferential reasoning that both negatively evaluated J’s actions for breaking the rule against self-endangerment and affirmatively justified F’s anger. A close sequential analysis of the M-K turn-taking reveals the chain of inferences that led to K’s moral evaluation that J was guilty. The objective of inferential thinking is the *truth*. It consists of passing from a premise or assumption considered as true to another judgment whose truth is believed to follow that of the former. When M asks why F is angry in T1, she assumes or states a premise in which *she believes* F is angry and implies that J is blameworthy. Thus, M acts epistemically in theorizing a truth claim. By asking why and expecting an answer, she shares her mind and seamlessly *transfers the premise* to K, asking her child to justify, validate, or give good reason in support of *her* belief and consequently *K’s belief* if he answers the question affirmatively–which he does. She has K provide the reason(s), thereby enabling him to participate empirically in the inference. K witnesses M’s claim in T2 in reporting that he has seen J “wandering around” in the forest.

M’s why questions concerning F’s subjective state stimulated K’s entry into the father character’s mind and aroused his own multiple *subjective* cognitive states that informed his reasoning: a *memory* of F in the movie responding angrily/vocally to J’s disobedience, an implicit *acknowledgment* and *agreement*, therefore, by K *evaluating* that F was, in fact, angry; an associated *observational memory* of J acting errantly in the forest; and, therefore, K participated in *moral reasoning* that causally and inferentially linked these memories to knowledge of breaking a cultural and family rule against putting oneself in harm’s way. These rich why questions are used with K so that he may comprehend the movie story as a whole and understand the connected relations between protagonists and the aroused mental states confirmed by neuroscience research (Decety and Howard, 2013). However, as Gallagher (2015) observes, such explanations are rarely used in maternal discourse with young children.

In T’s 3–4, M introduced a variation on the why-because pattern: these questions aim at revealing K’s nuanced evaluation of F’s anger–“angry in a good way or bad way?”–, and, potentially, at scaffolding K’s understanding of anger as an emotion containing underlying *motives*. We arrive at two suppositions: M is developing K’s emotional intelligence of anger as part of her instruction in the harm/care moral foundation and supports the prime role of affect in communicating about and making moral judgments. In response, K indicates that F’s anger is over-emotional because his observation that F’s “yelling” indicates that the boy finds F acting in a way that suggests the father is biased toward or being unfair to his daughter even though she is culpable. In Turns T14-T19, K’s responses further support his equivocal assessment of F’s motives in this context, for he also believes that the father’s yelling indicates he is “mad” (overly angry) at the reindeer. This line of questioning demonstrates that K has sufficient autonomy to come to his own assessment of F’s behavior. It also reveals that the boy does not yet understand that anger has a second primary objective in the harm/care moral foundation and that anger may also signify that F cares for and is protective of his daughter. As Graham et al. (2012) proposed, a moral foundation can be modified with experience.

After her preliminary use of the why-because pattern in inquiring into the reasoning and motives underlying F’s anger, M insists in T7 on bringing K around to her perspective by reiterating her theory and demands again that K answer why F was angry for his daughter’s wandering. M adds K’s prior observation of “wandering” into her scaffolding question. In response, K applies the moral foundational *principle*: “cause she wasn’t supposed to” (T8), i.e., that J’s behavior violates the harm/care norm, and this conclusion closes the chain of inferential reasoning that led K to *agree* to the truth of M’s claim. Now, they commit to the norm that harm, including potential harm to oneself, is unacceptable according to a cultural standard. As the why questions were repeated during this phase of the extended exchange (Ts 9–12), K was also scaffolded to imagine the consequences of J’s behavior (being hurt, shot, killed), and these statements strengthened the reasoning behind the joint M-K conclusion of the girl’s blame as violating the norm.

The pair’s conversational language provides support to findings that exposure to perspective-shifting discourse requiring children to adopt alternative perspectives on the same element of reality [the isomorphic reality of the movie] and sentential complement syntax [the grammatic turn-taking conventions we have described] may make independent contributions to improvements in theory-of-mind reasoning (Fernyhough, 2008, p. 33; Lohmann and Tomasello, 2003). While the present research agrees with this finding, we will expand the claim to state that in the paired I/RE mother–child, *why/because* conversation epistemic functions were stimulated in integrating K’s comprehension and judgments of the characters’ moral behaviors with the movie’s narrative information. We also conclude that each pair of turns in the why-because mechanism featured collaborative M/K theory-empirical roles that used audio-visual verbalizations (“yelling,” “wandering”) to generate moral attributes of the characters. These conversational roles and processes shared and integrated the pair’s memories of the movie.

#### 4.1.3 Sharing and recoding episodic/autobiographic memories into semantic understandings

The niche structure in which the viewing was succeeded by the conversation and use of the why-because pattern fostered a process whereby the participants drew upon their episodic memories of the movie events and reorganized them into verbally labeled comprehension of the characters and their moral attributes, potentially constructing new semantic memories. These constructive operations were probably conducted during the conversational retrieval through participants’ working memories involving their visual sketchpads and phonological loops (Baddeley, 2010). Thus, an external pattern, the why-because grammatical linguistic turn taking, interacted with internal episodic-semantic-working memory processes in constructing or reinforcing (there is no baseline measure to claim growth) K’s moral comprehension as instantiated in the characters’ moral attributes (harm/care; just/unjust; fair/unfair), as shown below (see 4.1.8). Such shared memories could solidify the separately experienced stories into “family stories,” resulting in a “team performance” in which neither party is the primary teller (Norrick, 2018), another perspective highlighting the productive functioning and instruction of cooperative norms supported in the SV conversation.

Afterward, we will show that the why-because epistemic tool generated “production units of meaning” (Magliano et al., 2013) based on the pair’s shared situational mental models of the movie narrative.

#### 4.1.4 Anger and moral evaluation processes

One of the skills that can be important to parenting is their capacity for adaptive emotion regulation, especially when they are distressed or angry in response to children’s misbehavior (Kopp, 1982; Morris et al., 2017). Angry expressions are known to curtail the behavior of others in situations where social rules or expectations have been violated (Averill, 1982), particularly in situations involving social hierarchy interactions, which parent–child interactions certainly are (Blair, 2003a; Blair and Cipolotti, 2000; Keltner and Anderson, 2000) (in Blair, 2005, p. 704). M’s placement of F’s anger at the outset of her why-because pattern was pivotal. Evolutionists have argued that breaches of the rule against placing oneself in danger are typically met with anger by family members and third-party adults (Burkart et al., 2018). Anger as an effect is a common trigger of the moral comprehension process in serving as a visceral assessment of, opposition to, and warning against norm breaking as wrong. Anger also serves other functions. Oatley and Duncan (1994, p. 54) suggests that “an emotion is triggered by a noticeable event and at its core is a change of readiness for action as the significance of the event is evaluated in relation to the person’s concerns.” The process typically includes a conscious feeling such as happiness, anger, fear, or disgust.” In this context, anger serves as an ancestral emotion program that originated to resolve conflicts in favor of the concerns of the angry individual (Sell et al., 2009). “When the anger program detects that the other party is not placing ‘sufficient’ weight on the welfare of the actor, anger is triggered [and]…places more weight on the welfare of the angry individual” (Sell et al., 2009, p. 15074). However, when observing that F went for his gun in his initial viewing of the movie, K considered that J evaluated her father’s behavior as expressing “bad” anger, and the boy also interpreted this action as being “mad” at the deer. And, when F “yelled,” K evaluated his expression as “bad” anger. To reinforce *her* meaning of anger, later in the conversation (T21), M forcefully argued that F was being protective and afraid that J could be hurt “wandering around in the snow.” Thus, moral understandings of norm-breaking and corrective actions are communicated through negative emotions like anger and fear as integrated in the father-daughter relationship and the M/K participants’ empathic relationship, following, as would be predicted by a process-relational moral theory (Carpendale et al., 2021).

#### 4.1.5 Empathic communicative interactions between M and K and F and J in conversational knowledge building

The initial and sustained focus through repeated questioning in the conversational interactional mechanism on explaining/evaluating F’s anger served to invoke K’s empathic communications with his mother and, we propose, further amplified his movie-driven empathic feelings for and identification with the movie characters. As achieved in developmentally mature children (*ca.* 4+ years), empathy is the capacity to be affected by and recognize the emotional states of others, particularly in distressful situations, to assess the reasons for these states, and to identify with the perspective of others (Decety and Jackson, 2004; Decety and Meyer, 2008; Eisenberg, 2000; Hoffman, 2001). Empathy has been identified as a phylogenetically ancient capacity essential for regulating cooperative social interactions and the foundation for altruistic, prosocial, and moral behaviors such as sharing with and helping others (de Waal, 2008, p. 281–282). Initially, empathy was conceived only as an emotional reaction in one party to the affective state of another party (Hoffman and Saltzstein, 1967) in order to assume the perspective and role of another person, thereby enabling prediction of their intentions, motives and likely actions (Feshbach and Feshbach, 1987). However, empathy increasingly refers to cognitive operations in sharing emotions. “Perspective taking is also treated as a part of empathy when it leads to emotion sharing or caring for others (e.g., Decety, 2011; Zaki, 2014)” (in Wondra and Ellsworth, 2015, p. 411). Blair (2005, p. 699) claims that empathy is, in fact, synonymous with the most frequently researched cognitive developmental capacity over the past half century: “Cognitive empathy is effectively theory of mind.”

Empathic operations were prominent in driving the intricate mechanisms of the SV conversation as they supported the understanding of the fictional characters in the *Prancer* narrative. “According to appraisal theory, empathy is possible whenever an observer [e.g., M] appraises a target’s situation [e.g., K’s]. If the observer appraises the target’s situation the same way as the target, empathy occurs” (Wondra and Ellsworth, 2015, p. 418). When M asked K why F was angry, she questioned whether K shared her view that J was responsible for causing her father’s emotions. In K’s response, it was clear that he agreed to hold J responsible because he observed that she wandered in the forest. As a follow-on effect, M’s questions appear to scaffold K’s empathic affective identification with F’s anger and J’s blame for harming her father. Cognitive empathy was evident in M and K’s mind-sharing and mindreading of the story characters that involved the boy’s developed theory of mind. M shared her mind with her son; he needed to read her mind to understand her questions, and she induced him to mindread F’s anger. In sum, M and K were practicing *empathic scaffolding* in their conversation concerning J, but not as we have observed concerning F’s anger at the deer.

#### 4.1.6 Space–time coordination: mental time travel and conversational script replacement of the historical narrative

To reiterate, in text comprehension models, the reader forms a situational schema in which events are causally linked within a narrative time and space in much the way that we understand real-world events, but this schema needs to be supported by an ability to “time travel.” “[N]arrative allows humans to extend their temporal horizon; it is the ability to travel forward and back in time that makes narrative fundamentally different from communicative non-narrative events that are limited to the immediate present” (Ferretti, 2019, p. 5). In Ts 1–2 the exchanges establish J’s wandering in the forest as the *spatial* setting for danger and therefore the site where the harm/care norm was broken, thereby causing the father-daughter conflict. As part of harm/care moral socialization, M signifies to K that the forest is dangerous, particularly during hunting season at dusk.

M’s reference to F’s anger (T1) also changed the *temporal* sequence by starting the conversation with F’s angry behavior that historically occurs in a scene later, toward midway in the movie, to induce K to empirically witness and causally link J’s errant actions in the forest scene that occurs in the opening of the movie. It is conjectured that M intended to direct and focus K’s attention on the harm F suffered in seeing his daughter in danger and to induce her son to flashback to J’s specific act of misconduct. By accomplishing this flashback and later in the conversation by scaffolding the boy to imagine the future consequences of breaking the norm, K demonstrates his capability for Mental Time Travel (MTT), a “faculty that allows humans to mentally project themselves backward in time to re-live or forward to pre-live events” (Tulving, 2002; see also Suddendorf and Corballis, 2007, p. 299; Ferretti et al., 2017).

M’s strategy is comparable to conventions in many crime stories in film and literature in which the *fabula*, a formal literary criticism term referring to the historical sequence of events, is reorganized in the conversation by the *syuzhet*, a term referring to the plot (Propp, 1928/1968; Shklovsky, 1917/1965). In crime stories, a genre that *Prancer* only represents analogously, the writer typically begins with the crime (J’s violation), and the reader/viewer is invited to flashback and work alongside the “detective” or “witness” (K’s observation of the “crime” in the forest) (Nicholson, 2017). More generally, stories gain interest by placing the crisis first, then flashing back. In the movie “Citizen Kane.”

[T]he fabula is the story of Kane’s life, from birth to death. The syuzhet, on the other hand, starts with Kane’s death [the crisis] and continues as the story of a journalist investigating Kane’s life, interspersed with a series of flashbacks (Nicholson, 2017, p. 1).

Concerning the deer, K’s assessment of F’s motives was likely influenced by the boy’s prior knowledge obtained through his initial viewing of the movie in which the father went for his gun intending to shoot the animal. While this occurred at the end of the movie, K apparently concluded that this meant that F was “mad” at the deer throughout the story as it was recapitulated during the conversation. He used the information, erroneously, to infer that F was biased against/acting unfairly toward the deer. Thus, both M and K used MTT to structure their moral arguments in negatively evaluating J’s and F’s respective behaviors.

#### 4.1.7 Causal structures, causal talk, and children’s comprehension

Narratives compress and encode the causal relations between events over time and the planning and sequencing of goal-directed actions embedded in cultural environments (Graesser et al., 1997). “Herman (2013, p. 237) affirmed from a structural standpoint, one of the hallmarks of narrative is [the] linking of phenomen[a] into causal-chronological wholes” (Ferretti, 2019, p. 5). In T2, K’s response that identified J’s “wandering” thereby causally linked the girl’s behavior to her father’s anger. Peterson and Slaughter (2003) found the frequency of reported use of causal, explanatory talk by mothers to their 4–5-year-old to be most helpful in predicting children’s social understanding. Donaldson (2003) suggest that causal connectives might serve to direct children’s attention to specific causal relationships.

#### 4.1.8 Character identification attributes: a scaffolding tool for judging characters’ moral attributes

Across the discussion, the SV pair’s joint and individual moral judgments were elaborated and accumulated into Character Identification Attributes (CIAs), including various descriptions of F’s angry behavior and J’s morally condemnable unsafe actions. NEST proposes that throughout the conversation, these CIAs supported K’s identification with characters and understanding of the movie events as a whole. According to Cohen (2001, 251), “identification is a process in which there is temporary replacement with heightened emotional and cognitive connections with a character and is a response to textual features.” Linguistic cues involved in sociocultural transfer induce various conceptual structures of identification that enable researchers to measure the degree of identification with characters during communications (Krieken et al., 2017, p. 2; Cohen, 2006). The why-because turn taking induced K’s moral understanding of the characters as well as revealing M’s moral understanding. As they acquired verbal moral attributes, the father and daughter characters served as *scaffolded repositories* for K’s affective/moral comprehension of the events in the movie story. By co-authoring their feelings and motives, the CIAs likely enabled K to identify empathically with the characters and recall the moral lesson when he retold the story.

### 4.2 Father’s CIA: anger attributes

Following is a sequential assemblage of the several pairs of turns forming F’s CIA with the speaker in parentheses [The anger attributes are interactively related to the violation of moral norms as indicated in brackets].

(M) F is angry. [Harm/Care].
(K) F is “bad angry” (yelling). [Fairness].
(M) F is angry (second attribution). [Harm/Care].
(M & K) The pair tacitly concludes that F is right and justifiably angry because he responds appropriately to the violation of the rule against self-endangerment. [Harm/Care].
(K) F is “mad” (very angry) at the deer. [Fairness].
(M) F is “good angry”: protective of and fearful for J. [Harm/Care].
(K) “Everyone was yelling”- J yelling at F and F yelling at J. [Fairness].
(K) “That, that she [J] was yelling at F” (negatively evaluating F’s intentions toward the reindeer). [Fairness].

Concerning the father’s character, M justified his anger by inducing K to agree that J had broken the rule against unsafe behavior, causing harm to her father. M also defended F against K’s claim that the father was mad at the deer, arguing that the dad was “good angry,” i.e., protective of his daughter. M instructed K that anger comprises both positive and negative motives. From a child discipline perspective, it may be inferred that M wants K to identify with and justify F’s role in the conflict as categorically against unsafe behavior.

While K agrees with M about the daughter’s misbehavior, the boy is unwilling to support F’s treatment of the reindeer. K offers descriptive attributions that negatively evaluate the father’s *motivations* underlying his anger. From K’s perspective, F’s anger is overly strong (yelling), reflecting the boy’s understanding that the tonic strength of emotion is critical to its intention; K also takes F’s yelling when their paths cross in the forest to indicate that the dad is overreacting to J’s misconduct and that, perhaps, he is being unfair to and biased against his daughter. According to K, F’s unfair motivation in this context is also indicated because he is “mad” at the deer, which is hardly a reason to kill the animal from the child’s perspective, and this speaks to F’s possible characterological disposition to be aggressively angry and, hence, unfair, in general. F’s CIA indicates that K, perhaps in imitation of and/or competition with M, is able to independently structure a prosecutorial inquiry into the father’s moral character, just as M has done for the daughter’s moral character.

### 4.3 J’s CIA

The harm/care and fairness/justice judgments were also elaborated and accumulated into attributions identifying J’s moral character. Following is an assemblage of pairs of turns forming J’s CIA.

(K) J acts dangerously (“wandering around”) [Harm/Care].
(K) J is wrong/guilty for violating the rule against putting oneself in harm’s way (“because not supposed to”). [Harm/Care].
(K) J could suffer consequences of misbehavior: “could be hurt, shot, killed” [Harm/Care].
(K) “That, that she [J] was yelling at F” for going for his gun to harm Prancer. [Fairness].

Thus, K agrees with M’s implied accusation that J violated a norm, but also that F may also have violated a norm in wanting to harm the deer and that the father made the situation more dangerous by going for his gun as well as yelling.

## 5 Narrative ecological scaffolding: K’s retold story

“The girl was in the forest and saw the footprints. Then the dad was going shopping and was really yelling at her in the truck. She should not have done that, but then the reindeer came back. Everyone was yelling. But he was saved!”

While K’s retold story is condensed, it does include the historical space–time-causal chain sequence of scenes/events in the movie: girl in the forest revealing her goal, looking for signs of reindeer; father’s arrival and his goal (shopping); demonstration of conflict between F and J (yelling); deer’s return and more conflict/fighting (mutual yelling); deer’s salvation. K’s narrative includes the father’s angry condemnation of J’s actions (“really yelling at her”), the boy’s knowledge of the rule (“she should not do that”), and his understanding of J’s strong objection to F’s intentions to shoot the deer (her yelling is part of “everyone yelling”). The story concludes with a happy outcome (“saved”). K’s retold story is conventional in that it recounts the developmentally normative causal chain for a 5-year-old, and the visible goal-directed behaviors shown in the movie, but is exceptional in expressing the critical moral judgment: “She should not do that.”

While some other retold stories in the extended exchange group were longer and better formed (see Clarke-Stewart and Beck, 1999, p. 409–410), K’s story is singular in extracting the story’s moral and likely in advance of developmental norms (Walker and Lombrozo, 2017). No other retold story in this corpus contained the harm/care moral applied to people. In their review of story comprehension research, Baker and Stein (1978, p. 18) concluded that “recall difficulties do not necessarily reflect failures to comprehend.” Other stories in the corpus all focused on the harm to the reindeer, several of the remedies to which consisted of charming but faulty reasoning, such as bringing the animal to a deer hospital or suggesting that “grandfather” could help the deer as he had helped the family dog by attaching a splint.

## 6 Discussion

### 6.1 The cooperative mechanisms of scaffolding: measuring the degrees of joint communication and common ground in mother–child pairs

The present study is compatible with and supports extensive research in both scaffolding and joint attention that mother–child mutual coordinated focus on shared cultural objects means that children “can learn not just *from* the other but *through* the other…children must come to understand why, toward what outside end, the other person is using the tool or symbol” (Tomasello, 1999, p. 6). By *through the other* we interpret K’s coordinated participation with his mother during the conversation. Because evidence of K’s level of moral comprehension in the conversation far exceeded his ability to communicate his competency in his retold story, we pursue the development of a method for measuring the pair’s cooperative levels of scaffolding as the degrees to which there is mother–child joint attention and direction for generating children’s moral comprehension of a narrative through epistemic knowledge construction processes. Siposova and Carpenter (2019) provide criteria for assessing the degree to which interlocutors are engaged in high-level intentional joint attention while sharing mutual objects, including the degree to which they share common goals and prior knowledge. They call for experimental designs in which the participants can only succeed if they are engaged in high attention and common knowledge (Siposova and Carpenter, 2019, p. 34), criteria met in the present experiment. An important aspect of common knowledge is that it refers to assumed shared information because they are grounded in some form of joint experience (Bohn and Köymen, 2018). The *Prancer* movie in the recursive experimental niche provided this shared source of experience.

Interlocutors with common knowledge have a mutual belief that the listener has understood the speaker’s reference (Clark and Wilkes-Gibbs, 1986), and this is compatible with the SV pair’s why-because tool, for they have.

knowledge and application of how and when to use utterances appropriately that combines with grammatical knowledge (of semantics, syntax, morphology, phonology) in the production of utterances to generate a coherent text comprehensible to its intended audience (Allan, 2013, p. 7).

Joint attention theory also presumes that the potential for shared understanding gains additional support when a pair has “common ground” (Clark and Wilkes-Gibbs, 1986). Common ground refers to participants’ shared presuppositions based on shared cultural background knowledge (Segerberg, 1973).

During scaffolding, common ground refers to common knowledge and beliefs mutually held by the interlocutors, including shared situational schemata of the utterance and world spoken of during conversations and scenarios, such as the harm/care scenario in the movie. “[O]ur brains look for, detect, and store structured patterns of information that constitute part of ‘common knowledge’” (Allan, 2013, p. 4). The discourse processes in the SV Protocol demonstrated that the SV pair jointly constructed several forms of common ground concepts:

#### 6.1.1 Shared episodic and semantic memories/knowledge

The pair demonstrated a shared intentional focus on their episodic memories of the movie and jointly reorganized them with the verbal descriptive language of the characters in the conversation, integrating their semantic lexicon knowledge (Baddeley, 2010).

#### 6.1.2 Narrative situational mental models

The pair jointly attended to the protagonists by representing their causally related actions in the movie’s space and time.

#### 6.1.3 Harm/care and fairness/justice moral foundations

The pair co-created judgments of the father and daughter characters’ harm/care and fairness moral attributes, as shown in the CIAs and K’s enacted procedural justice roles (discussed below).

#### 6.1.4 Why-because mechanism

During the SV conversation, the pair’s coordinated use of the why-because mechanism demonstrated the pair’s joint attention and shared narrative comprehension of the key dimensions of moral events in the story: participants’ goals, sequential order of events, violations of the canonical; use of the harm/care norm in judging the violation; inclusion of the narrator’s perspective [the screenplay writer and director] as well as viewing the story from the perspective of the protagonists (Bruner, 1990, p. 77; Mar, 2018a,b). The conversation also displayed the participants’ shared internal capabilities, including mutual recursive mindreading of the protagonists’ moral actions in the movie, their shared empathic understanding of anger as disapproval, and they used second person conversational language augmented by coordinated third person references to the F and J characters (Siposova and Carpenter, 2019, p. 5).

#### 6.1.5 Emotion norms: anger

The pair shared the common cultural meaning of anger as disapproval and opposition. M and K also demonstrated their joint empathic understandings as they agreed on their mutual interpretation of J’s moral responsibility for causing F’s anger.

However, M and K *did not share common ground* concerning F’s intentions and motives toward his daughter and the deer. In citing his yelling and going for his gun, K’s accusation of F’s motives showed his lack of development, both in misunderstanding the positive protective meaning of anger and the rule about hurting wild animals. M and K did, however, have coordinated and practiced joint language in inquiring into good and bad ways of expressing emotions. It can be argued that M’s questions opened the way for K’s disagreement about F precisely because she wanted to help her son comprehend the rule of moral comprehension and his still emerging knowledge of motives in ascertaining responsibilities.

### 6.2 Summary: evolution of cooperative scaffolding in a mother–child pair

Experimental evolutionary development engineering concepts and tools were tested in this study, including a recursive ecological niche and the selection of a mother–child pair that demonstrated cooperative scaffolding fitness. The SV pair constructed a high developmental standard of mother–child cooperative scaffolding, measured as the degrees to which there is mother–child joint attention and direction for generating children’s moral comprehension of a family conflict. Recently, innovative hyperscanning methods were used to measure interpersonal neural synchronization in interpersonal communications. These measures of neural coupling in brain activity could provide confirmatory data of shared representations and interpersonal predictive coding using both phonological and non-verbal visual signals during pair discourse processes, demonstrating mutual understanding (Jiang et al., 2021).

Several researchers have argued that justice and other cultural institutions should be considered part of the scaffolding system (Odling-Smee, 2010; Crisafi and Gallagher, 2009; Gallagher, 2013; Sperber and Hirschfeld, 2004; Gillespie and Cornish, 2010; Weaver et al., 2004; Burkart et al., 2018; Beck and Wood, 1993). We look to a model of cultural representation as a tool in socialization to explain M’s agency in her interactive exchanges with K. These agents adapted various cultural institutions, for example, education, justice, and emotions, to represent situations; this “presupposes cultural values, meanings, and assumptions [that]…culture is infused in our ability to represent the world” (Christopher and Bickhard, 2007, p. 271).

Narrative ecological scaffolding theory claims that the evolutionary developmental engineering method successfully adapted contemporary and ancestral cultural institutions and practices into the pair’s cooperative scaffolding tools. The why-because engine, for example, concerned the use of narrative inquiry (I/RE), an instructional method adapted from *education/tutoring* in the earliest universities and now employed in contemporary classrooms. The *movie constituted a contemporary cultural technology,* providing a young child with an accessible, emotionally heightened moral conflict. The investigation of the father character’s *anger addressed an ancestral emotion* theorized as evolving to negotiate conflicts in favor of the angry person. By having to explain F’s anger, K was induced to provide weight and justification to the father’s emotion. Although K’s comprehension of anger was sufficient to justify F’s condemnation of J’s violation, his immature understanding led him to misjudge F’s motives toward the reindeer. The conversation also assumed the form of a mother–child joint *investigation* of the conflict, with the mother’s questions theorizing the father’s legitimate response to norm-breaking and the child supplying empirical observations and *justice procedures* to research and judge the characters. The justice cultural system may be elaborated on in the following way because it provides the most holistic model for representing the SV pair’s scaffolding as it incorporates other cultural institutions.

The justice legal system comprises a ubiquitous cultural institution whose procedural methods have been engrained in worldwide households, at the least through exposure to police and courtroom procedures, e.g., *Law and Order, LA Law*, etc., and *Judge Judy* type programs on television, or, unhappily, for many families through direct exposure to law enforcement and the courts. Cultural representations of law enforcement and justice could provide a ready-made set of procedures for analogical use in the moral socialization of children. The SV scaffolding could be interpreted as such by institutional, cultural standards because J’s behavior could easily be viewed as a kind of law-breaking “violation,” albeit a misdemeanor, contrary to family rules and hence subject to investigations, undoubtedly directed by M in this case, but ably assisted by K as apprentice investigator.

Applying this perspective, the SV conversation demonstrated that M induced K to practice quasi-legal methods and justice roles to support her theory of J’s wrongdoing. K *witnessed* J’s wandering in the forest (T2) thereby providing evidence that empirically supported M’s theory; the boy cross-examined and prosecuted F’s *motives in questioning the father’s capacity as judge* of his daughter and the deer by observing, respectively, his yelling and going for his gun; as a juror, he *judged J’s culpability according to the law,* “Cause she wasn’t supposed to,” (T8) thereby holding her accountable according to the harm/care rule; and, he warned J of the dire *consequences* of breaking the rule: “because she could have got shot” (T12); afraid she was gonna die” (T20). By the end of the discussion, J is identified by her mother and son as guilty of causing *harm,* as shown in her CIA. In this procedural justice perspective, the SV conversation supports the claim that people, including 5-year-old, think and act more like intuitive lawyers (investigators and mock trial participants) than scientists (Ditto et al., 2009).

The cooperative epistemic scaffolding relationship of the SV pair may be best characterized as mother–child co-investigators and prosecutors of moral norm-breaking violations generating situational information enabling the participants to judge the story characters. In effect, the law and its procedures serve as a moral script for judging and adapting to breaches of norms.

### 6.3 Developing a NEST application: a scaffolding training program using the zone of proximal development

Having established a high standard of cooperative epistemic scaffolding for moral comprehension in the SV pair, how can its methods be transferred to comparable mothers and their late pre-K, K, and early school-aged children? An outline of a prospective scaffolding training program is developed from the SV scaffolding tools and the ZPD (Vygotsky, 1978; John-Steiner, 1985). The SV cooperative standard consists of a why-because conversational tool that directs the mother–child pair’s epistemic processes concerning shared common ground concepts in comprehending moral foundations. The concepts comprise narrative comprehension mental models (schema), including protagonists’ conflicting goals and causal relations in the space/time of the story, the moral signaling function of characters’ emotions, and procedural justice roles. Selected ZPD features taken from the SV study may be used recursively to visualize and systematize these SV epistemic processes.

The ZPD is a model that bridges the distance between a child’s actual and potential development through imitation of the structural relations of a problem through collaborative mother–child communications. The ZPD operationalizes teaching and learning in a specific sociocultural material environment; the mind is conceived as working through cultural artifacts and shared perspectives. In the present case, it is the moral mind (mental model) whose processes are modeled as working through recursive narrative genres–movie, conversation, retelling–under the guidance of a cooperative mother–child pair. Our approach is to first train the mothers to operate in the ZPD to support their understanding of movie structures, then for them to scaffold their children.

The NEST: ZPD is conceived/visualized as follows: the Zone is the material artifact landscape of the movie story; in recursive viewings of the 5′ movie clip, each mother crosses the ZPD under the instructor’s guidance. Variations of the why-because tool are applied consecutively after each of the four crossing passages to assess the mothers’ understanding of (1) the harm/care and fairness/justice moral foundations, (2) the father/daughter conflicting goals, (3) the anger and fear emotions, and (4) justice procedures. For example, after the 1st viewing, the instructor uses why questions to assess each mother’s comprehension of the aforesaid moral issues in the movie. Each recursive crossing then builds on the previous crossing. After the second viewing, the instructor questions comprehension of the characters’ goals as they contribute to their moral conflict; after the third viewing, the instructor questions the purpose and impact of each character’s anger and fear in moral communications. After the fourth viewing, the instructor questions comprehension of the justice procedures, in particular the mother–child functions as co-investigators in judging the characters. Independently, the mothers participating in the ZPD crossings review selections from the present article, including the movie description, its affordances, and the SV conversational protocol. In a fifth viewing, the instructor scaffolds each mother using the exact why-because tool of the SV conversational protocol. Later, each trained mother scaffolds her child, and the results are compared with the SV standard.

## Data Availability

The original contributions presented in the study are included in the article/supplementary material, further inquiries can be directed to the corresponding author.
